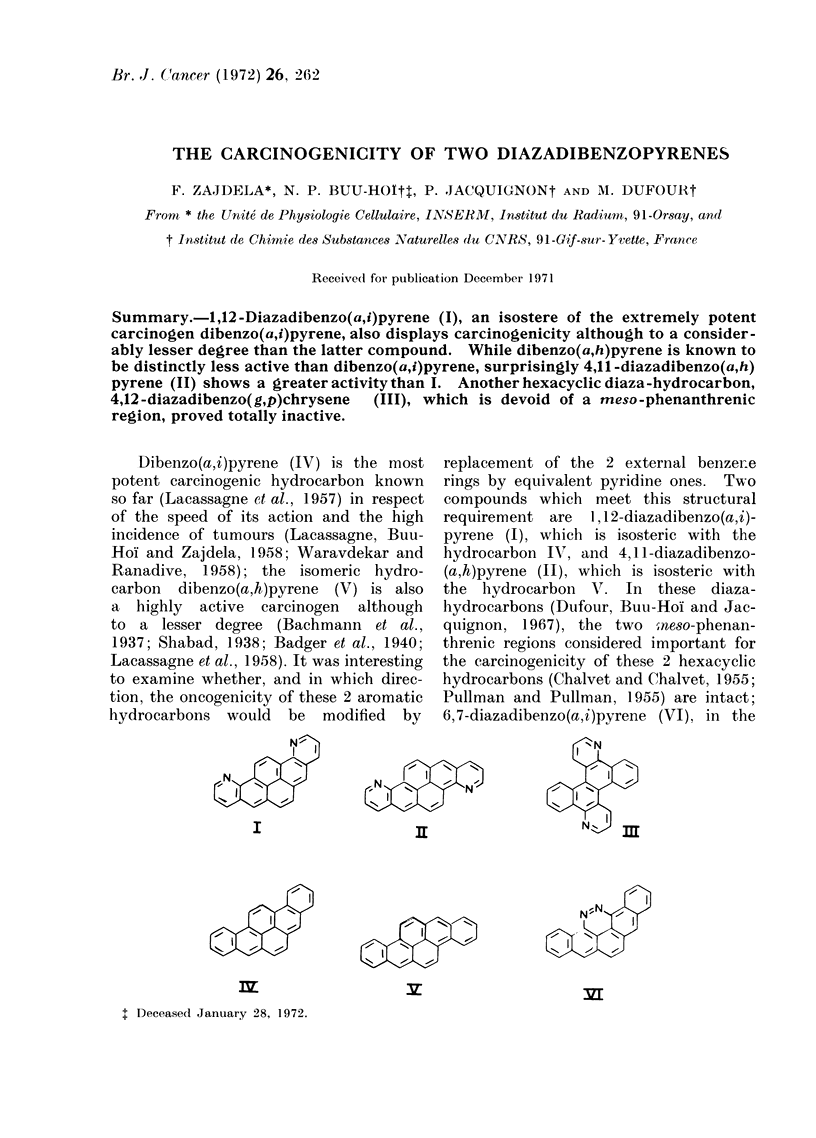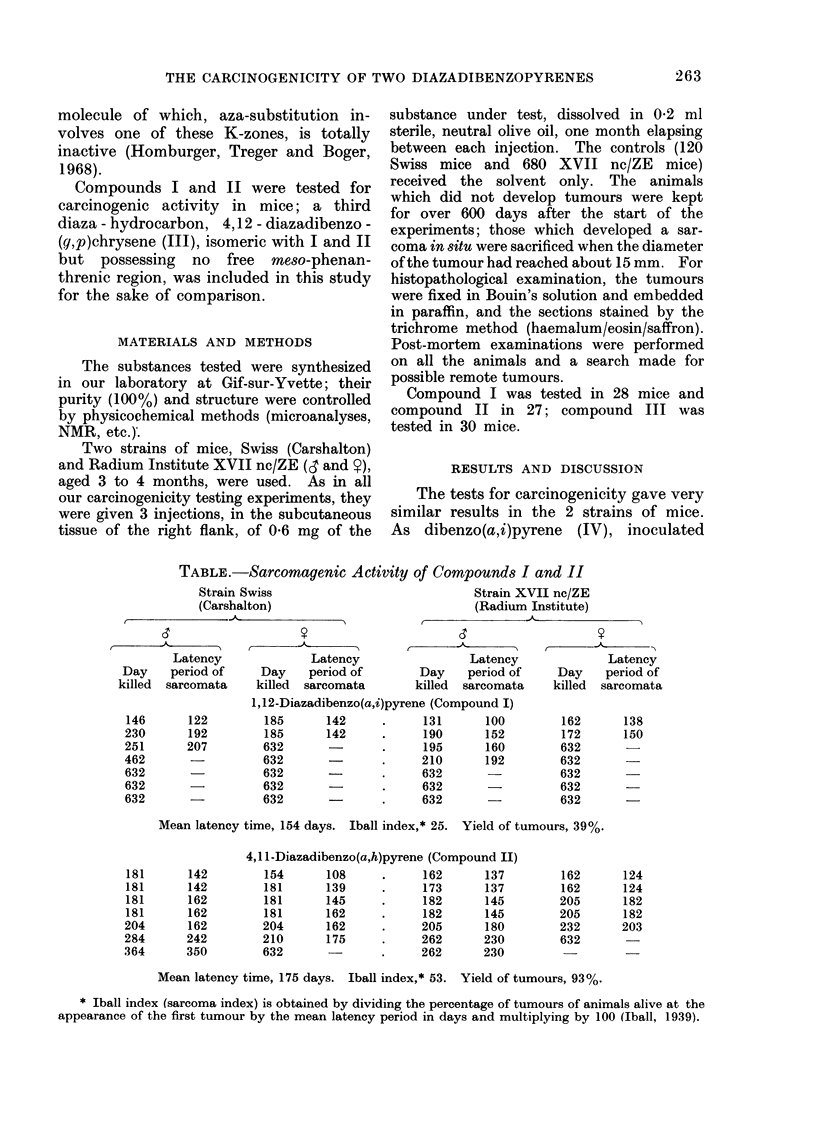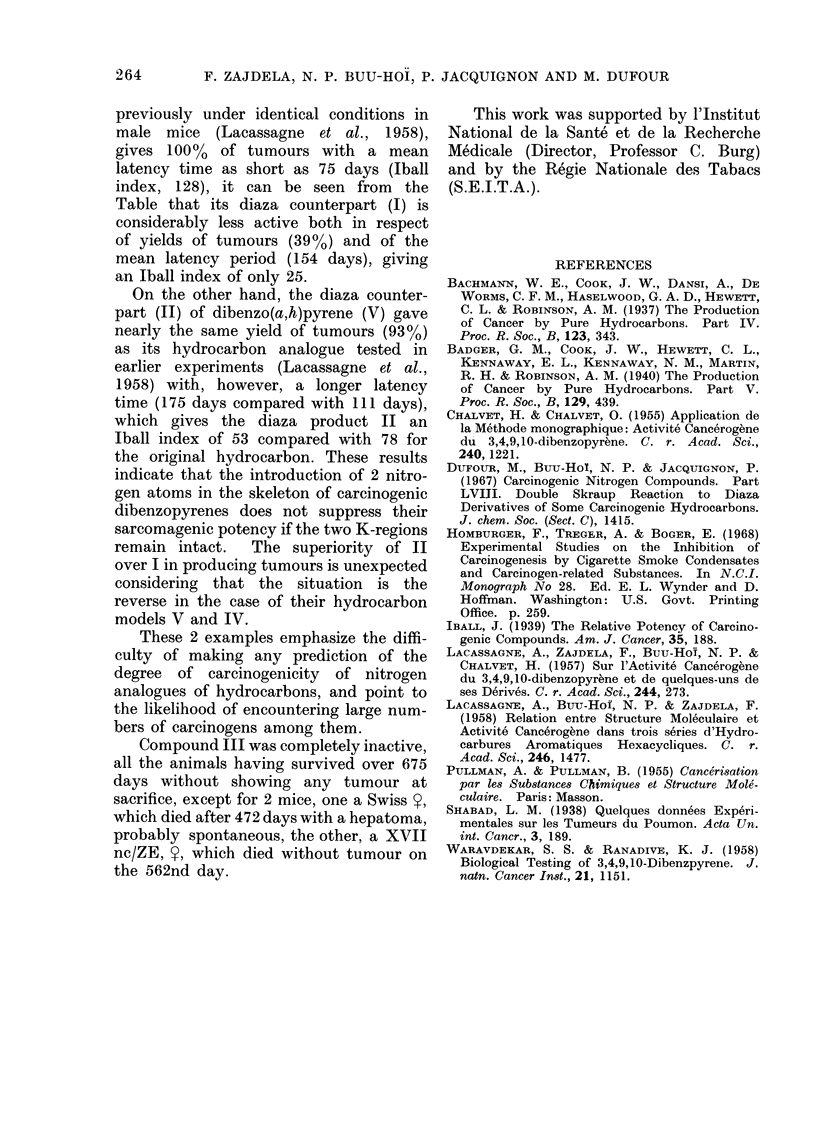# The Carcinogenicity of Two Diazadibenzopyrenes

**DOI:** 10.1038/bjc.1972.35

**Published:** 1972-08

**Authors:** F. Zajdela, N. P. Buu-Hoï, P. Jacquignon, M. Dufour

## Abstract

1,12-Diazadibenzo(*a,i*)pyrene (I), an isostere of the extremely potent carcinogen dibenzo(*a,i*)pyrene, also displays carcinogenicity although to a considerably lesser degree than the latter compound. While dibenzo(*a,h*)pyrene is known to be distinctly less active than dibenzo(*a,i*)pyrene, surprisingly 4,11-diazadibenzo(*a,h*)pyrene (II) shows a greater activity than I. Another hexacyclic diaza-hydrocarbon, 4,12-diazadibenzo(*g,p*)chrysene (III), which is devoid of a *meso*-phenanthrenic region, proved totally inactive.


*Br. J. Cancer* (1972) **26**, 262

### THE CARCINOGENICITY OF TWO DIAZADIBENZOPYRENES

F. ZAJDELA*, N. P. BUU-HOÏ†‡, P. JACQUIGNON† AND M. DUFOUR†

*From * the Unité de Physiologie Cellulaire, INSERM, Institut du Radium,* 91-*Orsay, and*

† *Institut de Chimie des Substances Naturelles du CNRS,* 91-*Gif-sur-Yvette, France*

Received for publication December 1971

**Summary.—1,12-Diazadibenzo(*a,i*)pyrene (I), an isostere of the extremely potent carcinogen dibenzo(*a,i*)pyrene, also displays carcinogenicity although to a considerably lesser degree than the latter compound. While dibenzo(*a,h*)pyrene is known to be distinctly less active than dibenzo(*a,i*)pyrene, surprisingly 4,11-diazadibenzo(*a,h*) pyrene (II) shows a greater activity than I. Another hexacyclic diaza-hydrocarbon, 4,12-diazadibenzo(*g,p*)chrysene (III), which is devoid of a *meso*-phenanthrenic region, proved totally inactive.**

Dibenzo(*a,i*)pyrene (IV) is the most potent carcinogenic hydrocarbon known so far (Lacassagne *et al.*, 1957) in respect of the speed of its action and the high incidence of tumours (Lacassagne, Buu-Hoï and Zajdela, 1958; Waravdekar and Ranadive, 1958); the isomeric hydrocarbon dibenzo(*a,h*)pyrene (V) is also a highly active carcinogen although to a lesser degree (Bachmann *et al.*, 1937; Shabad, 1938; Badger *et al.*, 1940; Lacassagne *et al.*, 1958). It was interesting to examine whether, and in which direction, the oncogenicity of these 2 aromatic hydrocarbons would be modified by replacement of the 2 external benzene rings by equivalent pyridine ones. Two compounds which meet this structural requirement are 1,12-diazadibenzo(*a,i*)-pyrene (I), which is isosteric with the hydrocarbon IV, and 4,11-diazadibenzo-(*a,h*)pyrene (II), which is isosteric with the hydrocarbon V. In these diaza-hydrocarbons (Dufour, Buu-Hoï and Jacquignon, 1967), the two *meso*-phenanthrenic regions considered important for the carcinogenicity of these 2 hexacyclic hydrocarbons (Chalvet and Chalvet, 1955; Pullman and Pullman, 1955) are intact; 6,7-diazadibenzo(*a,i*)pyrene (VI), in the

I

II

III

IV

V

VI

‡ Deceased January 28, 1972.

molecule of which, aza-substitution involves one of these K-zones, is totally inactive (Homburger, Treger and Boger, 1968).

Compounds I and II were tested for carcinogenic activity in mice; a third diaza-hydrocarbon, 4,12-diazadibenzo-(*g,p*)chrysene (III), isomeric with I and II but possessing no free *meso*-phenanthrenic region, was included in this study for the sake of comparison.

### MATERIALS AND METHODS

The substances tested were synthesized in our laboratory at Gif-sur-Yvette; their purity (100%) and structure were controlled by physicochemical methods (microanalyses, NMR, etc.).

Two strains of mice, Swiss (Carshalton) and Radium Institute XVII nc/ZE (♂ and ♀), aged 3 to 4 months, were used. As in all our carcinogenicity testing experiments, they were given 3 injections, in the subcutaneous tissue of the right flank, of 0·6 mg of the substance under test, dissolved in 0·2 ml sterile, neutral olive oil, one month elapsing between each injection. The controls (120 Swiss mice and 680 XVII nc/ZE mice) received the solvent only. The animals which did not develop tumours were kept for over 600 days after the start of the experiments; those which developed a sarcoma *in situ* were sacrificed when the diameter of the tumour had reached about 15 mm. For histopathological examination, the tumours were fixed in Bouin's solution and embedded in paraffin, and the sections stained by the trichrome method (haemalum/eosin/saffron). Post-mortem examinations were performed on all the animals and a search made for possible remote tumours.

Compound I was tested in 28 mice and compound II in 27; compound III was tested in 30 mice.

### RESULTS AND DISCUSSION

The tests for carcinogenicity gave very similar results in the 2 strains of mice. As dibenzo(*a,i*)pyrene (IV), inoculated

TABLE.—*Sarcomagenic Activity of Compounds I and II*

| Strain Swiss (Carshalton) | | | | Strain XVII nc/ZE (Radium Institute) | | | |
|---|---|---|---|---|---|---|---|
| ♂ | | ♀ | | ♂ | | ♀ | |
| Day killed | Latency period of sarcomata | Day killed | Latency period of sarcomata | Day killed | Latency period of sarcomata | Day killed | Latency period of sarcomata |
| 1,12-Diazadibenzo(*a,i*)pyrene (Compound I) | | | | | | | |
| 146 | 122 | 185 | 142 | 131 | 100 | 162 | 138 |
| 230 | 192 | 185 | 142 | 190 | 152 | 172 | 150 |
| 251 | 207 | 632 | — | 195 | 160 | 632 | — |
| 462 | — | 632 | — | 210 | 192 | 632 | — |
| 632 | — | 632 | — | 632 | — | 632 | — |
| 632 | — | 632 | — | 632 | — | 632 | — |
| 632 | — | 632 | — | 632 | — | 632 | — |
| Mean latency time, 154 days. Iball index,* 25. Yield of tumours, 39%. | | | | | | | |
| 4,11-Diazadibenzo(*a,h*)pyrene (Compound II) | | | | | | | |
| 181 | 142 | 154 | 108 | 162 | 137 | 162 | 124 |
| 181 | 142 | 181 | 139 | 173 | 137 | 162 | 124 |
| 181 | 162 | 181 | 145 | 182 | 145 | 205 | 182 |
| 181 | 162 | 181 | 162 | 182 | 145 | 205 | 182 |
| 204 | 162 | 204 | 162 | 205 | 180 | 232 | 203 |
| 284 | 242 | 210 | 175 | 262 | 230 | 632 | — |
| 364 | 350 | 632 | — | 262 | 230 | — | — |
| Mean latency time, 175 days. Iball index,* 53. Yield of tumours, 93%. | | | | | | | |

* Iball index (sarcoma index) is obtained by dividing the percentage of tumours of animals alive at the appearance of the first tumour by the mean latency period in days and multiplying by 100 (Iball, 1939).

previously under identical conditions in male mice (Lacassagne *et al.*, 1958), gives 100% of tumours with a mean latency time as short as 75 days (Iball index, 128), it can be seen from the Table that its diaza counterpart (I) is considerably less active both in respect of yields of tumours (39%) and of the mean latency period (154 days), giving an Iball index of only 25.

On the other hand, the diaza counterpart (II) of dibenzo(*a,h*)pyrene (V) gave nearly the same yield of tumours (93%) as its hydrocarbon analogue tested in earlier experiments (Lacassagne *et al.*, 1958) with, however, a longer latency time (175 days compared with 111 days), which gives the diaza product II an Iball index of 53 compared with 78 for the original hydrocarbon. These results indicate that the introduction of 2 nitrogen atoms in the skeleton of carcinogenic dibenzopyrenes does not suppress their sarcomagenic potency if the two K-regions remain intact. The superiority of II over I in producing tumours is unexpected considering that the situation is the reverse in the case of their hydrocarbon models V and IV.

These 2 examples emphasize the difficulty of making any prediction of the degree of carcinogenicity of nitrogen analogues of hydrocarbons, and point to the likelihood of encountering large numbers of carcinogens among them.

Compound III was completely inactive, all the animals having survived over 675 days without showing any tumour at sacrifice, except for 2 mice, one a Swiss ♀, which died after 472 days with a hepatoma, probably spontaneous, the other, a XVII nc/ZE, ♀, which died without tumour on the 562nd day.

This work was supported by l'Institut National de la Santé et de la Recherche Médicale (Director, Professor C. Burg) and by the Régie Nationale des Tabacs (S.E.I.T.A.).

### REFERENCES

Bachmann, W. E., Cook, J. W., Dansi, A., De Worms, C. F. M., Haselwood, G. A. D., Hewett, C. L. & Robinson, A. M. (1937) The Production of Cancer by Pure Hydrocarbons. Part IV. *Proc. R. Soc., B*, **123**, 343.

Badger, G. M., Cook, J. W., Hewett, C. L., Kennaway, E. L., Kennaway, N. M., Martin, R. H. & Robinson, A. M. (1940) The Production of Cancer by Pure Hydrocarbons. Part V. *Proc. R. Soc., B*, **129**, 439.

Chalvet, H. & Chalvet, O. (1955) Application de la Méthode monographique: Activité Cancérogène du 3,4,9,10-dibenzopyrène. *C. r. Acad. Sci.*, **240**, 1221.

Dufour, M., Buu-Hoï, N. P. & Jacquignon, P. (1967) Carcinogenic Nitrogen Compounds. Part LVIII. Double Skraup Reaction to Diaza Derivatives of Some Carcinogenic Hydrocarbons. *J. chem. Soc.* (*Sect. C*), 1415.

Homburger, F., Treger, A. & Boger, E. (1968) Experimental Studies on the Inhibition of Carcinogenesis by Cigarette Smoke Condensates and Carcinogen-related Substances. In *N.C.I. Monograph No* 28. Ed. E. L. Wynder and D. Hoffman. Washington: U.S. Govt. Printing Office. p. 259.

Iball, J. (1939) The Relative Potency of Carcinogenic Compounds. *Am. J. Cancer*, **35**, 188.

Lacassagne, A., Zajdela, F., Buu-Hoï, N. P. & Chalvet, H. (1957) Sur l'Activité Cancérogène du 3,4,9,10-dibenzopyrène et de quelques-uns de ses Dérivés. *C. r. Acad. Sci.*, **244**, 273.

Lacassagne, A., Buu-Hoï, N. P. & Zajdela, F. (1958) Relation entre Structure Moléculaire et Activité Cancérogène dans trois séries d'Hydrocarbures Aromatiques Hexacycliques. *C. r. Acad. Sci.*, **246**, 1477.

Pullman, A. & Pullman, B. (1955) *Cancérisation par les Substances Chimiques et Structure Moléculaire.* Paris: Masson.

Shabad, L. M. (1938) Quelques données Expérimentales sur les Tumeurs du Poumon. *Acta Un. int. Cancr.*, **3**, 189.

Waravdekar, S. S. & Ranadive, K. J. (1958) Biological Testing of 3,4,9,10-Dibenzpyrene. *J. natn. Cancer Inst.*, **21**, 1151.